# A new hypothesis: some metastases are the result of inflammatory processes by adapted cells, especially adapted immune cells at sites of inflammation

**DOI:** 10.12688/f1000research.8055.1

**Published:** 2016-02-16

**Authors:** Leili Shahriyari

**Affiliations:** 1 Mathematical Biosciences Institute, The Ohio State University, Columbus, OH, USA

**Keywords:** Metastasis, Cancer, Chronic inflammation, Adapted immune cells, Inflammatory processes, Immune system, Platelets, Wound healing process.

## Abstract

There is an old hypothesis that metastasis is the result of migration of tumor cells from the tumor to a distant site. In this article, we propose another mechanism for metastasis, for cancers that are initiated at the site of chronic inflammation. We suggest that cells at the site of chronic inflammation might become adapted to the inflammatory process, and these adaptations may lead to the initiation of an inflammatory tumor. For example, in an inflammatory tumor immune cells might be adapted to send signals of proliferation or angiogenesis, and epithelial cells might be adapted to proliferation (like inactivation of tumor suppressor genes). Therefore, we hypothesize that metastasis could be the result of an inflammatory process by adapted cells, especially adapted immune cells at the site of inflammation, as well as the migration of tumor cells with the help of activated platelets, which travel between sites of inflammation.  If this hypothesis is correct, then any treatment causing necrotic cell death may not be a good solution. Because necrotic cells in the tumor micro-environment or anywhere in the body activate the immune system to initiate the inflammatory process, and the involvement of adapted immune cells in the inflammatory processes leads to the formation and progression of tumors. Adapted activated immune cells send more signals of proliferation and/or angiogenesis than normal cells. Moreover, if there were adapted epithelial cells, they would divide at a much higher rate in response to the proliferation signals than normal cells. Thus, not only would the tumor come back after the treatment, but it would also grow more aggressively.

Many cancers arise from sites of chronic inflammation ^1^. Immune cells inside the chronic inflammation site initiate tumor progression by releasing reactive oxygen or nitrogen species, which lead to DNA damage in epithelial cells ^2^. Inflammation not only can cause mutation in epithelial cells ^3^, but can also change their fitness ^4^.

In chronic inflammation, T-cells might become adapted to send high levels of proliferation signals, and regulatory T-cells might have been changed to prevent their inhibition ^5^. Effector T-cells also create an environment for tumor initiation and progression by releasing tumor-promoting cytokines IL-6 ^2^.

These findings suggest that cells at the site of chronic inflammation are adapted to the wound healing process. Immune cells are adapted to send signals of proliferation or angiogenesis, and tissue cells are adapted to proliferation (like inactivation of tumor suppressor genes). These adaptations lead to the initiation of a tumor.

If there are adapted immune cells, then we can look at metastasis from a new perspective. Any site of inflammation might recruit adapted immune cells, and then they contribute to the inflammatory process there. If immune cells are adapted to send more signals of proliferation and angiogenesis, then new tumors would initiate at sites of inflammation. Below we gather some evidence that supports this hypothesis.

• There is a hypothesis that metastasis is the result of cancer cell migration from the tumor extracellular matrix to the bloodstream or lymphatic vessels as circulating tumor cells (CTCs), and then to the new site. CTCs only use lymphatic routes to migrate to the nearby lymph nodes, but not for traveling long distances ^6^. Cell migration occurs when tumor epithelial (E) cells lose their cell-cell adhesion and become motile mesenchymal (M) cells, i.e. epithelial-mesenchymal transition (EMT). When these CTCs exit the bloodstream, they undergo a reverse process called mesenchymal-epithelial transition (MET) to continue their differentiation and develop a secondary tumor ^7– 9^.
  In inflammatory breast cancer, there is a correlation between immune activation and CTCs with EMT characteristics ^10^. Cohen *et al.* hypothesized that EMT could be the result of inflammatory processes initiated by activated immune cells ^10^. Epithelial cells from the colonic epithelium of patients with benign colon diseases also circulate in the blood ^11^. During wound healing, epithelial cells migrate and disperse as individual mesenchymal cells by down regulating cell-cell junctions ^12^. Thus, CTCs in blood could be the result of inflammation.
• By querying published available data sets ^13– 17^, we calculate the probability of not detecting any CTCs in blood from patients with metastatic breast cancer, and the result is 0.6. That means there might be other phenomena, beside CTCs, that cause metastasis. Since, no CTCs were detected in the blood of 29% of metastatic breast cancer patients starting a first or new line of therapy, it is unlikely that treatments are responsible for not observing CTCs in blood ^16^.
• CTC-clusters are rare compared with single CTCs, however CTC-clusters, which usually include platelets and white blood cells ^18^, significantly increase metastatic potential ^19^. Moreover inhibition of platelet activation or platelet depletion decreases metastasis rates ^20– 22^. There is a hypothesis that metastasis is reduced when the host is platelet depleted because platelets are protecting tumor cells from natural killing cells ^23, 24^ Platelets in tumor cell clusters release transforming growth factor *β* (TGF- *β*) ^25^, and tumor cells that are bound to platelets highly express EMT-associated genes ^26^.
• Cancer of unknown primary origin (CUP) is defined by the presence of metastatic disease with no identified primary tumor. CUPs are 3.5% of all human malignancies, and one of the ten most frequent cancers worldwide ^27^. The major sites of CUP are bones, liver, lung, and lymph nodes ^28^, the sites where immune cells are most active.
• New studies show that recruited bone marrow progenitor cells change the host’s environment to generate the “pre-metastatic niche” to which the tumor cells metastasize ^29^. Bone marrow-derived haematopoietic progenitor cells (HPCs) that express vascular endothelial growth factor receptor 1 form cellular clusters at pre-metastatic sites before appearance of a single tumor cell ^30^.
• There is evidence of metastasis to the site of injury. Two patients with squamous cell carcinoma of the lung developed distant localized metastatic disease at sites of physical injuries; one to the knee injured in an accidental fall six weeks earlier, and the other to portions of the liver injured in a mechanical fall two months earlier ^31^. In a mice model of metastatic breast cancer, radiation-induced pulmonary injury lead to chronic inflammatory responses, and development of pre-metastatic niches ^32^. In another mice model, hepatic ischemia-reperfusion injury increased the number of liver metastases of human pancreatic cancer (Capan-1) cells, which were injected into the mice spleen ^33^. Several studies show that lung injury induced by the chemotherapy drug, bleomycin, increases lung metastases; they also observed tumor cell adherence to extracellular matrix and fibrin at injured areas ^34, 35^. Therefore we suggest that the sites of injuries are potential metastatic sites.

We hypothesize that chronic inflammation can cause adapted bone marrow derived cells (for example, adapted macrophages or T-cells) and/or adapted tissue cells (for instance, adapted epithelial cells or stromal fibroblasts) to lead tumor initiation and progression. If adapted immune cells are present, then the new site of inflammation might recruit these adapted immune cells and cause metastasis. Additionally, the new site of inflammation may recruit activated platelets. The activated platelets travel between sites of inflammation, including the site of inflammatory carcinoma. Tumor cells can link to adhesion receptors on platelets and travel to the new site of inflammation. The activated platelets start the wound healing process at the new site, which now includes some tumor cells. As the tumor cells respond to the wound healing signals more strongly than normal cells, new tumors would initiate in the site of inflammation ( Figure 1).

**Figure 1.**
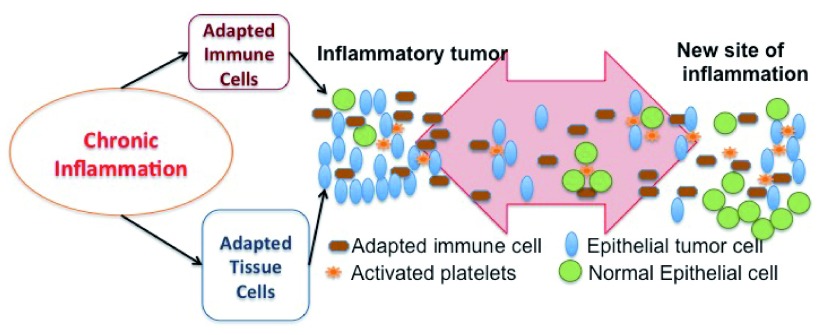
Metastasis. Chronic inflammation leads to adapted tissue and/or adapted immune cells. These adaptations cause tumor initiation. A new inflammation site recruits these adapted immune cells. The adapted immune cells start the wound healing process at the inflammation site and send more inflammatory signals than normal immune cells. Thus, a new tumor initiates there. Also, the activated platelets travel between sites of inflammation, including the site of primary inflammatory tumor. Tumor cells can link to adhesion receptors on platelets and travel to the new site of inflammation with the help of platelets. The activated platelets start the inflammatory process at the new site, which now includes some tumor cells. Tumor cells respond to the inflammatory signals more strongly than normal cells. The transported tumor cells initiate a tumor at the inflammation site.
