# Supplementary material for: A new hypothesis: some metastases are the result of inflammatory processes by adapted cells, especially adapted immune cells at sites of inflammation
Source: F1000Res. 2016 Feb 16;5:175. [Version 1] doi: 10.12688/f1000research.8055.1 (PMC4847566; doi:10.12688/f1000research.8055.1)
Supplement: Peer review history

# Peer review: Referee response for version 1

**DOI:** 10.5256/f1000research.8666.r13490

**Published:** 2016-04-27

**Authors:** Alain Borgeat

**Affiliations:** 1 Orthopedic University Hospital Balgrist, Zurich, Switzerland

There is more and more evidence that cancer metastases and inflammation share a common pathway. Therefore this hypothesis has a relevant scientific background. Prospective clinical trials looking at this issue will be welcome.

I have read this submission. I believe that I have an appropriate level of expertise to confirm that it is of an acceptable scientific standard.

# Peer review: Referee response for version 1

**DOI:** 10.5256/f1000research.8666.r13492

**Published:** 2016-04-22

**Authors:** Patrice Forget

**Affiliations:** 1 Department of Anesthesiology, Université Catholique de Louvain, Brussels, Belgium

The inflammatory nature of cancer, and especially metastases development, becomes a paradigm shift. This text summarizes well some new hypotheses and future challenges. Next steps to translate this in clinical trials are then needed.

I have read this submission. I believe that I have an appropriate level of expertise to confirm that it is of an acceptable scientific standard.

# Peer review: Referee response for version 1

**DOI:** 10.5256/f1000research.8666.r13279

**Published:** 2016-04-18

**Authors:** Maryam Keshtkar Jahromi

**Affiliations:** 1 Division of Infectious Diseases, Department of Medicine, Johns Hopkins University School of Medicine, Baltimore, MD, USA

This hypothesis has a potential to make a positive impact in cancer research and drug discovery. For cancer patients, it might help to accelerate both the development of promising therapies and the potential of personalized medicine — treatments based on an individual’s unique immune deficiency. This is a very interesting and unique idea which needs more research.

I have read this submission. I believe that I have an appropriate level of expertise to confirm that it is of an acceptable scientific standard.
